# Physiological Mechanisms of Plant Growth-Promoting Rhizobacteria in Enhancing Abiotic Stress Tolerance of Vegetable Crops: A Review

**DOI:** 10.3390/plants15050686

**Published:** 2026-02-25

**Authors:** Jinyong Yang, Mingshan Tang, Hongjiao Zhao

**Affiliations:** College of Advanced Agricultural Sciences, Ningbo Summit Advancement Discipline of Biological Engineering, Zhejiang Wanli University, Ningbo 315100, China; yangjinyong117@163.com (J.Y.); 13435686725@163.com (M.T.)

**Keywords:** vegetables, abiotic stresses, plant growth promoting rhizobacteria (PGPR)

## Abstract

Global climate change is increasing the impacts of abiotic stresses on plants. Vegetables are rich in vitamins, minerals, dietary fiber, and a variety of phytochemicals, and thus, are of great significance to human health. The growth of vegetable crops is regulated by a variety of abiotic stress factors, which not only affect their normal growth and metabolism but also lead to reduced yield and quality. Plant growth-promoting rhizobacteria (PGPR) can modulate the morphological or physiological characteristics of plants via nitrogen fixation, phosphorus dissolution, potassium dissolution, production of siderophores, secretion of secondary metabolites and hormones, and induction of plant stress resistance gene expression. This consequently increases the nutrient utilization rate in plants, improving their yield, quality, and stress resistance. In this review, the literature focused on how rhizosphere growth-promoting bacteria can improve the resistance of vegetable crops to drought, extreme temperature, heavy metals, and salt stresses is reviewed, and relevant application prospects and research directions provide a reference for further research on stress resistance and strategies to increase the yield of vegetable crops.

## 1. Introduction

Against the background of global climate change and the deterioration of the ecological environment, the pressures faced by plants—such as drought, salinity, and heavy metal pollution—are becoming increasingly serious. These factors seriously limit plants’ normal growth and development, thus posing major threats to agricultural production and ecological balance [1,2,3]. Vegetables are an essential food for humans, providing a variety of nutrients (e.g., vitamins and minerals) that are necessary for the human body [4]. The World Health Organization (WHO) recommends a daily intake of 400 g of fruits and vegetables to meet the body’s micronutrient requirements and to prevent noncommunicable diseases [5], with commonly consumed vegetables including cucumber (*Cucumis sativus* L.), tomato (*Solanum lycopersicum* L.), watermelon (*Citrullus lanatus*), and cabbage (*Brassica oleracea* L.). During their growth cycle, vegetable crops are often affected by a variety of abiotic stresses, resulting in decreased yield and quality [6,7]. To enhance the adaptability of these crops to adverse conditions, researchers have explored a variety of methods, such as strengthening agricultural infrastructure, improving field management, and genetic improvement of crop varieties [8,9,10]. In recent years, as a potential biological resource, plant growth-promoting rhizobacteria (PGPR) have attracted increased research attention because they can improve the stress resistance of plants through metabolites [11]. Studies have shown that inoculation with specific microorganisms can effectively alleviate the inhibitory effects of drought on vegetable growth, reduce oxidative stress damage, and improve root architecture [12,13]. Some PGPR strains of the genus *Bacillus* are capable of releasing volatile organic compounds, which play an important role in plant-microorganism interactions, thus promoting plant growth and enhancing their tolerance to drought, salt and mineral deficiency stresses [14,15]. For example, in cucumber, *Bacillus zanthoxyli* HS1 induced a reduction in stomatal aperture, promoted callose accumulation in the cell wall, and enhanced the activities of enzymes such as superoxide dismutase and catalase by releasing VOCs, thereby mitigating the adverse effects of heat and salt stress on plants [16]. However, PGPR-induced systemic resistance is often limited by plant species, growth stages, and environmental factors. Therefore, it is still of great significance to continuously explore new beneficial strains and their active metabolites. To date, the positive effects of PGPR on the growth and yield of vegetable crops have been widely verified, with the associated mechanisms involving the regulation of volatile substances, alterations in endogenous hormone levels, improvement of nutrient absorption efficiency, and enhancement of stress resistance [17]. In this article, we systematically reviewed the existing research on how rhizosphere microorganisms improve the abiotic stress resistance of vegetable crops focusing on the effects of PGPR under stress conditions and the associated physiological mechanisms. With this study, we hope to provide theoretical support for the application of microbial technology in the stress-resistant cultivation of vegetable crops.

## 2. Effects of Abiotic Stress on the Growth and Production of Vegetable Crops

**Figure 1:**
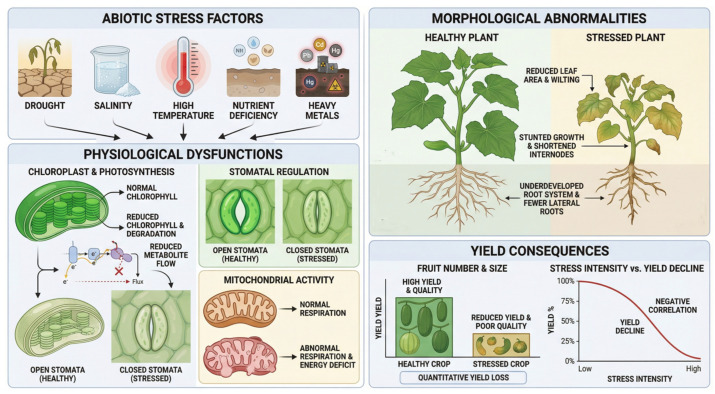
Effects of Abiotic Stress on Vegetable Crops. Abiotic stress can induce significant alterations in the morphological structure of crops, including leaf area reduction, plant height inhibition, and root dysplasia. Simultaneously, physiological functions are compromised, as evidenced by decreased photosynthetic rates, reduced assimilate partitioning, and altered respiration rates, ultimately leading to substantial yield losses.

For normal growth, vegetable crops require sufficient light, water and suitable temperature conditions. In nature, these crops are highly susceptible to a variety of abiotic stress factors, among which drought, salinity, and extreme temperatures are the main factors affecting their physiological and biochemical processes. Abiotic stress can lead to significant changes in the morphological structure of crop plants, including leaf area reduction, plant height reduction, and root dysplasia [18,19]. Additionally, stress can negatively affect the physiological functions of plants, typically manifested as a decreased photosynthetic rate, a reduction in the formation of assimilation products, and an abnormal respiration rate [20,21], eventually leading to a serious reduction in crop yield (Figure 1).

Vegetable crops are characterized by distinct attributes that influence their responses to abiotic stress and its consequences: (I) They exhibit high water and nutrient demands due to their rapid growth and succulent tissues, rendering them particularly sensitive to water and osmotic stresses [22]. (II) Intensive cultivation practices, such as frequent irrigation and elevated fertilizer inputs, can exacerbate issues related to salinity and heavy metal accumulation [23]. (III) The direct harvesting of vegetative or reproductive organs (leaves, fruits, roots) for human consumption means that stress-induced morphological changes (e.g., alterations in leaf size and fruit shape) and the accumulation of anti-nutritional compounds (e.g., nitrates, heavy metals) can significantly affect marketable yield and food safety [24]. (IV) Furthermore, the quality parameters of vegetables (including color, texture, flavor, and phytochemical content) are highly influenced by abiotic stresses [25,26].

### 2.1. Drought Stress

Drought is one of the important environmental factors affecting agricultural production, posing a serious threat to global food security. Under drought conditions, plants suffer a decrease in cell osmotic pressure due to cell water loss, which hinders the absorption of water and nutrients [27]. Leaves may shrink, curl, or even turn yellow, and the plant’s physiological functions will also be disturbed. If the water shortage continues, plants will face irreversible damage, which may eventually lead to death [28]. Vegetable crops are particularly sensitive to drought under which they reduce water transpiration through stomatal closure, while also limiting the absorption of carbon dioxide, resulting in significant decreases in the photosynthetic rate, transpiration rate, and stomatal conductance. Additionally, the synthesis of chlorophyll is inhibited and decomposition accelerates, further weakening the plant’s ability to complete photosynthesis [29]. Second, drought can cause membrane lipid peroxidation, thus increasing the permeability of cell membrane; this leads to electrolyte leakage and, ultimately, structural and functional damage to the cell membrane [30]. A water deficit also leads to reduced cell turgor pressure, the blockage of multiple enzymatic reactions, disordered metabolic pathways, the accumulation of harmful substances, and decreased energy utilization efficiency, ultimately inhibiting plant growth and reducing yield and quality [31]. In addition, drought can also affect the development and maturation of fruits, resulting in reductions in yield and the accumulation of sugars, nutrients, and flavor-related compounds, thus reducing the economic value of fruits and vegetables, and causing significant losses for agricultural producers [32]. For leafy vegetables such as lettuce and spinach, drought-induced reductions in leaf area and cell turgor directly translate to lower fresh weight [33]. In fruit plants such as tomato and pepper, water deficits during flowering and fruit set disrupt carbohydrate partitioning, leading to blossom-end rot or misshapen fruits, severely compromising quality [34].

### 2.2. Salt Stress

Soil salinization has become a major obstacle to the sustainable development of global agriculture [35]. In vegetable crops, the high-permeability environment formed due to high concentrations of sodium and chloride ions hinders the absorption of water and nutrients by the roots, leading to the excessive accumulation of these ions in cells [36]. Different crops and their varieties present significant differences in salt tolerance and thresholds [37]. Studies have shown that salt stress has significant inhibitory effects on the physiological activities of vegetable crops—including seed germination, water absorption, cell elongation, and lateral branch formation—at multiple growth stages [38]. At the germination stage, an increase in the salt ion concentration leads to a decrease in the germination rate; furthermore, the root system presents a higher intracellular osmotic potential under salt stress, hindering the absorption of water, thus causing osmotic stress and physiological drought, which manifests as slow seedling growth, yellow leaves, root atrophy, or even death [39]. After salt ions enter the plant through the roots, they also inhibit photosynthesis and respiration, resulting in growth stagnation and abnormal morphology of the aboveground part [40]. In addition, a high-salt environment can induce the production of large amount of reactive oxygen species (ROS) in plants, and the subsequent generation of the lipid peroxidation product malondialdehyde (MDA) further destroys the cell membrane structure and interferes with physiological processes [41]. Salt stress can inhibit the activity of nitrate reductase, resulting in the accumulation of nitrate in vegetables (especially leafy vegetables such as spinach and lettuce), which poses a potential risk to human health [42]. In addition, salt stress can affect the metabolism of sugars (such as glucose) and organic acids (such as citric acid or malic acid) in plants such as tomato and strawberry, resulting in acid taste or flavor fading in their fruits, thus reducing their commercial value [43].

### 2.3. Other Stresses

Climate change has led to an increase in the frequency and intensity of extreme temperature events. Heat and cold stress have become important factors affecting the yield and quality of vegetable crops [44,45]. Temperature stress mainly interferes with photosynthetic function in plants by destroying the protein structure and lipid composition in the cell membrane, thus causing disordered physiological metabolism and inhibiting the normal growth and development of plant [46]. Vegetable crops are extremely sensitive to temperature changes. Continuous high temperatures have been shown to lead to decreases in the photosynthetic rate and photosynthetic pigment content in leaves, which seriously affects their production potential [47]. Moreover, for tomatoes and melons, high temperature conditions accelerate the activities of cell wall-degrading enzymes, resulting in rapid softening of fruits and poor tolerance to storage and transportation. Low temperature conditions affect starch conversion in cucumber, watermelon, and other fruits and vegetables, leading to abnormal sugar accumulation and, consequently, reduced sweetness and flavor. In addition, environmental stresses such as heavy metal pollution also restrict the growth and metabolism of vegetable crops by interfering with enzyme activities, destroying the cell ultrastructure and inducing oxidative stress [48,49]. Heavy metals can be enriched in the human body through the food chain, leading to chronic poisoning of the human body and causing irreversible damage to multiple systems and organs. Therefore, once the heavy metal content in the edible part of a plant exceeds the safety standard, it becomes “contaminated food.”

## 3. PGPR-Mediated Promotion of Vegetable Crops Growth Under Abiotic Stress Conditions

**Figure 2:**
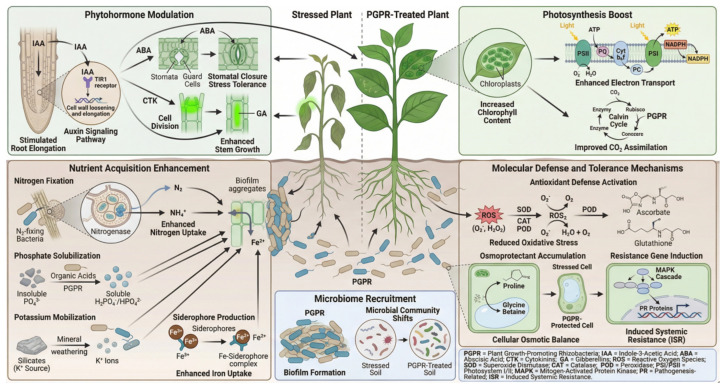
Schematic representation of plant growth-promoting rhizobacteria (PGPR)-mediated mechanisms for abiotic stress resistance in vegetable crops. Plants inoculated with PGPR exhibit growth-promoting attributes, such as the production of phytohormones (e.g., indole-3-acetic acid, IAA) and nitrogen fixation, which enhance stress tolerance through ACC deaminase activity. PGPR also induce the expression of stress-responsive genes, leading to the accumulation of various osmoprotectants and defensive compounds, as well as the detoxification of reactive oxygen species (ROS) within cells. The modulation of antioxidant activities helps to prevent cellular damage and maintain homeostasis. In addition, the recruitment of beneficial microorganisms contributes to enhancement of the microbial community structure in the rhizosphere soil. Collectively, these mechanisms improve growth, yield, and stress tolerance in vegetable crops.

The beneficial interplay between PGPR and plants under stress is orchestrated through a complex network of direct and indirect mechanisms, collectively termed Induced Systemic Tolerance (IST) [50,51], which operate synergistically to maintain plant homeostasis. The core mechanisms include the following: (1) nutrient acquisition enhancement via biological nitrogen fixation, phosphate and potassium solubilization, and siderophore-mediated iron chelation [52,53,54]; (2) phytohormone modulation, including biosynthesis of auxins (IAA), cytokinins, and gibberellins, or degradation of the ethylene precursor ACC by ACC deaminase (ACCD) to alleviate ethylene stress signaling [55,56]; (3) biochemical priming and osmoregulation, involving the production of bacterial exopolysaccharides (EPS) for soil aggregation and moisture retention, induction of plant antioxidant systems (SOD, CAT, POD, APX), and stimulation of plant osmolyte (proline, glycine betaine) biosynthesis [57,58]; (4) molecular and systemic signaling, such as the secretion of microbial volatile organic compounds (mVOCs) that can prime plant defense pathways and induce the expression of stress-responsive genes (e.g., DREB, RD29A, P5CS) [59]; and (5) rhizosphere engineering, where PGPR alter root exudation patterns and recruit other beneficial microbes to form a supportive, stress-resilient microbiome [60] (Figure 2).

At the molecular level, plant growth-promoting rhizobacteria influence phytohormone pathways by activating specific plant receptors and transcription factors; for instance, auxins produced by plant growth-promoting rhizobacteria bind to plant receptors, activating auxin response factors that regulate the expression of genes that are essential for root development and elongation [61]. Additionally, cytokinins produced by microbes interact with histidine kinase receptors on plant cell membranes, triggering response regulators that promote cell division and delay senescence, particularly under abiotic stress conditions [62]. Furthermore, microbial modulation of ethylene levels affects ethylene response factors, which mediate ethylene-induced signaling to enhance abiotic stress tolerance [63]. These molecular interactions between microbial signals and plant receptors optimize growth, improve nutrient acquisition, and strengthen stress resilience, even under challenging environmental conditions.

### 3.1. Plant Growth-Promoting Rhizobacteria (PGPR)

Plant growth-promoting rhizobacteria refer to a class of beneficial microorganisms that inhabit the soil surrounding the roots of plants and promote the growth of host plants. Common PGPR genera include *Bacillus*, *Pseudomonas*, *Flavobacterium*, *Azotobacter*, *Xanthomonas* and *Klebsiella* [64,65,66,67,68,69]. PGPR can promote the absorption and utilization of important nutrient elements such as nitrogen, phosphorus, and potassium in plants; they also have the ability to synthesize plant growth regulators such as IAA, which is conducive to the growth and development of plants and improves their yield and overall quality. In addition, PGPR also play an important role in enhancing plant stress resistance and disease control [70,71]; for example, in 1997, Bigirimana et al. were the first to report that Trichoderma can induce systemic resistance in plants, thereby enhancing the adaptability of crops to diseases and environmental stresses [72]. Additionally, studies have shown that *Burkholderia vietnamensis* B418 can effectively inhibit the harm inflicted by root-knot nematodes by regulating the microbial community structure in the root zone of watermelon [73]. Inoculation with *Pseudomonas fluorescens* improved the salt stress tolerance of vegetable crops [74]. PGPR have also been shown to be effective in the treatment of heavy metal stress; for example, *Acinetobacter pittii* enhanced the tolerance of plants to cadmium stress by up-regulating the expression of genes related to bacterial migration, amino acid metabolism, and carbon metabolism in the plant rhizosphere bacterial community and promoting cadmium mobilization in the rhizosphere soil [75]. Anjum et al. showed that inoculation of *Pseudomonas fluorescens* A506 enhanced the antioxidant activities of catalase, peroxidase, and superoxide dismutase; increased physiological traits such as chlorophyll content and proline content; and reduced the toxic effects of lead, thus alleviating lead stress in tomato [76].

### 3.2. Role of PGPR Against Drought Stress in Vegetable Crops

To date, a variety of PGPR strains such as *Pseudomonas*, *Bacillus*, *Burkholderia*, and *Acinetobacter* have been shown to enhance the drought resistance of plants [77]. For example, the *Flavobacterium* sp. GJW24 strain alleviated multiple negative effects of drought on cabbage by regulating stomatal closure and up-regulating the expression of drought stress-responsive genes such as *EXLB1*, *DREB2A*, *TIFY3a*, and *CSD3* (about 7.0-, 1.8-, 2.0-, and 1.9-fold, respectively) and maintained a 60% survival rate even under extreme water shortage conditions (water deficiency for 10 d) [78]. Similarly, the application of the *Bacillus subtilis* strain GOT9 in rapeseed activated the up-regulation of a variety of drought stress-related genes—especially *BrDREB1D*, *BrWRKY7*, and *BraCSD3*—involved in the dehydration response, thereby minimizing physiological damage. The enhanced expression of ABA-induced genes clearly indicated that GOT9 increased ABA accumulation in plants, thereby enhancing drought tolerance in rapeseed [79]. A PGPR strain of *Streptomyces* mitigated drought stress in tomato and regulated the expression of the transcription factors ethylene response factor 1 (ERF1) and WRKY70 [80]. Treatment of pepper with *Bacillus licheniformis* K11 led to up-regulation of the genes Cadhn, VA, sHSP and CaPR10, resulting in higher production of dehydrin-like protein, vacuolar H^+^-ATPase, small heat shock protein, and pathogenesis-related protein, which improved the survival of plants under severe drought conditions [81]. It has also been shown that *Pseudomonas aeruginosa* N5.12 and N21.24 conferred good drought resistance and stable yield in tomato, where the underlying mechanism was closely related to increases in photosynthetic pigments (chlorophyll a 70%, chlorophyll b 69% and carotenoids 65%), proline (33%), betaine (4.3%), and other substances, as well as increased expression of the abscisic acid synthesis rate-limiting enzyme gene *NCDE1* (9-cis-epoxycarotenoid dioxygenase; up-regulated about 2.7-fold) and the pyrroline synthase gene *P5CS* (Pyrroline-5-carboxylate synthase; up-regulated about 3.0-fold) [82]. In addition, Admassie et al. isolated drought-resistant bacteria from pepper (*Capsicum annuum* L.) roots under drought conditions, which were shown to promoted pepper root growth (the inoculated plants showed 41–79.6% higher root lengths, compared with the control) and alleviated drought stress damage through the production of extracellular polysaccharides and auxin [83]. In addition to adding a single strain, the drought resistance of plants can also be improved through the combined inoculation of rhizosphere growth-promoting bacteria; for example, the combined application of *Enterobacter hormaechei*, *Acinetobacter* sp., and *Pantoea dispersa* alleviated the damage in Indian mustard due to drought stress by enhancing osmotic regulation, reactive oxygen species (ROS) detoxification, and carbon and nitrogen metabolism. In addition, metabolic pathways related to starch and sucrose metabolism, galactose metabolism, and amino acid biosynthesis play key roles [84]. Moreover, plant growth-promoting rhizobacteria can also alleviate the inhibitory effects of drought stress on seed germination and seedling growth. Kumar et al. showed that the combined inoculation of *Bacillus amyloliquefaciens* NBRISN13 and *Pseudomonas putida* NBRIRA significantly promoted the germination and seedling growth of chickpea (*Cicer arietinum* L.) seeds under drought stress, compared with single-strain inoculation [85]; in particular, the bacterial consortium had a better effect on promoting drought resistance than either single inoculation, indicating better application prospects. These studies indicate that PGPR affect the expression of drought-related genes, triggering the production of various antioxidants, osmolytes, proline, and other important biomolecules that help to alleviate drought stress. In addition, several hormones produced by PGPR trigger a series of biochemical reactions in the host, enabling plants to better tolerate drought stress.

### 3.3. Role of PGPR Against Salt Stress in Vegetable Crops

Most vegetable crops are susceptible to growth inhibition under salt stress, which interferes with the morphological and physiological functions of crops, significantly limits the biomass of edible parts of plants, and leads to decreased yields. The harm to vegetables caused by salt stress is mainly due to the effects of osmotic water shortage, the accumulation of salt ions in the aboveground parts of plants, and imbalances in mineral nutrition, as well as their interactions. The roles that plant growth-promoting rhizobacteria play in alleviating salt stress have been confirmed in a variety of crops; for example, in eggplant (*Solanum melongena* L.), PGPR enhanced the activities of active oxygen scavenging enzymes (SOD and GR activities increased by 38.68% and 22.86%, respectively) and reduced the accumulation of sodium ions in roots (sodium ion content decreased by 38.44%), thus effectively improving the plants’ salt resistance (NaCl, 50 mM) [86]. As lettuce (*Lactuca sativa* L.) is a popular but salt-sensitive leafy vegetable, research on the salt tolerance of this crop has attracted significant attention. Ikiz et al. added PGPR biostimulants containing *Bacillus* and *Pseudomonas* to the nutrient solution used for lettuce hydroponic cultivation, and found that this not only significantly reduced the adverse effects of salt stress (NaCl, 50 mM) on plant biomass, plant height, leaf number and leaf area but also improved the absorption and utilization of N (22.3%), P (96.3%), K (82.1%), and other mineral elements in lettuce [87]. Additionally, rhizosphere growth-promoting bacteria can also alleviate the harm caused by salt stress in plants through the secreting of growth-promoting hormones; for example, *Bacillus subtilis* C8 promoted the growth and biomass of cucumber shoots (plant height increased by 47.58%, leaf area increased by 42%, dry weight increased by 34.97%) by secreting growth hormone IAA and producing siderophores, thus enhancing the plants’ salt tolerance (salinity level: 8 dS/m) [88]. Moreover, plant growth-promoting rhizobacteria can also produce extracellular polysaccharides, which have been shown to chelate Na^+^ (1563.5 mg/h), enhance hydroxyl scavenging activity (increased by 151.8%), and improve plant salt tolerance (salinity levels: 15.9 dS/m) [89]. In addition, the study of Khan et al. showed that *Pseudomonas fluorescens* NAIMCC-B-00340 and *Azotobacter chroococcum* Beijerinck 1901 reduced ethylene accumulation (decreased by about 80%) and enhanced antioxidant enzyme activities (SOD, CAT, APX, and GR increased by 58.40%, 25.65%, 81.08%, and 55.91%, respectively) in mustard (*Brassica juncea* L.) under salt stress (100 mM NaCl) through synthesizing ACC deaminase, thus maintaining reactive oxygen species homeostasis, and reducing the inhibitory effects of salt stress on plant growth [90]. Similarly, another ACC deaminase-producing endophytic strain, *Pseudomonas* sp. OFT5, conferred salt tolerance to tomato by reducing ethylene production [91]. Rhizobium inoculation has also been reported to control the expression of several ion affinity transporters; in this context, tissue-specific regulation of high-affinity K^+^ transporters (HKT) is critical for maintaining ion homeostasis in salt-stressed plant cells during plant-microbe interactions. Salt overload-sensitive genes (SOS) and other enzymes acting as sodium antiporters may help plants to adapt to salt stress. The salt-tolerant strains *Fonticola serratia* S1T1 and *Pseudomonas korea* S4T10 alleviated the effects of salt stress (200 mM) in cucumber. Microbial inoculants significantly improved the tolerance of cucumber to NaCl stress by up-regulating the expression of the ion transport genes HKT1 (1-2-fold), NHX (1-3-fold), and SOS1 (2-4-fold) [92]. In summary, PGPR can alleviate salt stress by improving soil health, nutrient absorption, hormone production, antioxidant activity, and the expression of stress response genes.

### 3.4. Role of PGPR Against Other Stresses in Vegetable Crops

High-temperature conditions inhibit the basic physiological functions of plants and lead to declines in crop yield in many agricultural regions around the world. As such, this problem has become the focus of global environmental attention. Recent studies have shown that plant growth-promoting rhizobacteria can enhance the resistance of many crops to high- and low- temperature stresses; for example, Chan et al. found that compared with uninoculated plants, the aboveground and root biomass (25.2% and 37.6%) and nutrients (e.g., nitrogen, phosphorus, potassium, calcium and iron; 33.3–217.1%) in lettuce were significantly increased when the crop was inoculated with heat-resistant *Klebsiella* sp. GRB10. GRB10 alleviated the adverse effects of heat stress (30 °C) on plants by regulating stomatal movement, improving photosystem II efficiency (15.0%), and reducing water consumption [93]. ACC deaminase and extracellular polysaccharides were shown to be synthesized by *Bacillus cereus* KTES under high-temperature conditions (42 °C), which improved the physiological and biochemical indices of tomato, including stem length, root length, fresh and dry weights, chlorophyll content, protein, proline (32.6–64.4%), and antioxidant activities (SOD, POD, and CAT increased by 42.9%, 96.2%, and 11.3%, respectively) [94]. The mixed flora composed of *Bacillus cereus* AR156, *Bacillus subtilis* SM21 and *Serratia* sp. XY21 was shown to promote the accumulation of soluble sugars, proline, and osmotic proteins in tomato, thus enhancing the antioxidant defense system and reducing osmotic stress in plant cells, enabling cold acclimation of seedlings and enhancing their cold tolerance (4 °C) [95]. In addition, the distinct role of *Streptomyces* sp. 506 (TOR3209) in enhancing cold stress tolerance in tomatoes is evident: the expression profile of stressed plants treated with TOR3209 indicated higher expression of ABA signaling genes mediated by HY5 (bZIP), including zeaxanthin epoxidase (ZEP1), 9-cis-epoxycarotenoid dioxygenase (NCED1), carotenoid dioxygenase, carotene beta-hydroxylase, and dehydrin (TASI4). In addition, TOR3209 diminished photosynthetic damage by adjusting the activities of the enzymes RUBISCO (Ribulose 1, 5-bisphosphate carboxylase/oxygenase), NAD-MDH, and NADP-MDH (malate dehydrogenases) [96]. These results indicate that the primary mechanism through which cold tolerance is conferred by this strain operates via the ABA pathway. Another study demonstrated that treatment of tomato plants with the psychrotolerant microbes *Pseudomonas vancouverensis* (OB155) and *Pseudomonas frederiksbergensis* (OS261) under chilling stress reduced the impact by enhancing proline levels and the activities of antioxidant enzymes such as superoxide dismutase (SOD), ascorbate peroxidase (APX), and glutathione (GSH) [97]. These studies illustrate that the majority of cold tolerance conferred by plant growth-promoting rhizobacteria (PGPR) is attributable to their interlinked effects on antioxidants, soluble sugars, proteins, proline, phenolics, phytohormones, and other related compounds. On the other hand, heavy metal pollution, as another major environmental pressure, poses multiple threats to both the sustainable development of agriculture and human health. The excessive accumulation of heavy metals in plants can inhibit their normal growth and development, while also causing public health risks through the food chain. Although some metal elements are essential for plant growth, they exhibit significant toxicity once they exceed critical concentrations. Bio-inoculants prepared with plant growth-promoting rhizobacteria (PGPR) have been shown to reduce the toxic effects of metals such as zinc, nickel, cadmium and antimony in vegetables including cucumbers, tomatoes, legumes, peppers and lettuce [98,99,100]. Liu et al. found that the inoculation of *Bacillus siamensis* R27 could significantly enhance the activities of antioxidant enzymes (SOD, CAT, POD increased by 27.9–31.7%) in lettuce, thus mitigating the reactive oxygen species (ROS) induced by Cd stress. Additionally, the expression of Cd transport-related genes such as *IRT1*, *Nramp1* and *HMA2* was down-regulated (by 71.4–87.5%), and the Cd^2+^ content in lettuce was reduced (by 38.9%), thereby reducing Cd^2+^ toxicity [101]. At present, only a limited number of studies have been conducted to identify the roles of key metal transporter/regulator genes and their functional regulation in PGPR-associated plants, and the detailed molecular mechanisms need to be further studied. Moreover, PGPR inoculation typically enhances the host plant’s antioxidant system in response to environmental stresses. Inoculation with *Bacillus* isolates improved the zinc tolerance of *Solanum tuberosum* by mitigating oxidative stress, as evidenced through an analysis of the expression levels of SOD, CAT, APX, DHAR, and GR genes [102]. Similarly, Khanna et al. observed that inoculation with plant growth-promoting rhizobacteria (PGPR) strains significantly enhanced the antioxidant system of Lycopersicon esculentum by up-regulating the mRNA expression of superoxide dismutase (SOD), peroxidase (POD), and polyphenol oxidase (PPO) genes under cadmium (Cd) stress [103]. In addition, Kang et al. showed that Lactobacillus MO1 enhanced tolerance to zinc stress and reduced metal toxicity by reducing hydrogen peroxide content and zinc absorption in cucumber roots and stems under zinc stress [104] (Table 1). In summary, rhizosphere growth-promoting bacteria have a good ability to alleviate the effects of abiotic stresses in vegetable crops; however, their application in the field needs to be studied further before their full commercialization. Many of the positive results reported thus far have been obtained in sterilized or low-microbial-background soil, which is far from the actual vegetable planting situation, potentially leading to a lack of replicability in real-world agricultural applications.

### 3.5. Field Application Effect and Challenges of PGPR Agent

Beneficial microbial inoculants promote plant growth through various mechanisms, including nutrient enhancement, production of plant hormones, secretion of siderophores, assembly of microbial communities, activation of native microbes, synthesis of antimicrobial compounds, and induction of plant resistance. Despite the compelling evidence obtained in controlled environments, the transition of PGPR from promising laboratory results to reliable, large-scale field application in the context of vegetable production faces significant hurdles. The very attributes that make vegetable systems productive—intensive management, high turnover, and focus on quality—also create unique challenges for microbial inoculants. Strains performing robustly in greenhouses often show diminished or inconsistent effects outdoors [105]. In particular, the application of a single microbial agent often faces problems such as functional limitations, poor colonization ability, and a low survival rate, attributed to the following: (I) difficulties in maintaining the viability and activity of bacteria during storage to achieve the desired effect in the field [106]; (II) poor rhizosphere colonization and persistence due to competition with established indigenous microbiota [107]; (III) environmental variability in soil pH, moisture, temperature, and organic matter [108]; and (IV) incompatibility with standard agronomic practices, such as the use of chemical fertilizers or pesticides that may inhibit bacterial viability [109]. Most commercial PGPR products have been developed for broad-acre crops such as cereals. Vegetable-specific formulations that consider differences in seed size (e.g., tiny lettuce seeds vs. large pumpkin seeds), planting methods (direct seeding vs. transplanting), and growth cycles are scarce. Effective delivery systems (e.g., seed coating, root dip for transplants, biofilm-based inoculants) tailored to vegetable production are needed [110]. The success of PGPR depends on their interactions with the native microbiome, which vary with the soil type, crop history, and genotype [111]. There is a paucity of data on how PGPR inoculants reshape the resident microbial community in vegetable rhizospheres and how this, in turn, influences plant health. The path to commercialization involves rigorous and costly regulatory processes for demonstration of safety and efficacy. For vegetable growers, the economic incentive must be clear—PGPR application should reliably translate into quantifiable improvements in yield, quality (size, color, shelf-life), and resource use efficiency (water, fertilizer) to justify its adoption. Therefore, exploring more effective strategies to improve the efficiency and safety of microbial inoculants has become an urgent need for the sustainable development of vegetable production systems.

## 4. Conclusions and Perspectives

Plant growth-promoting rhizobacteria (PGPR) regulate the physiological metabolic network of host plants under abiotic stress through multi-dimensional mechanisms [112]. The core of PGPR is to construct a mutually beneficial “plant–microorganism” functional consortium. On the one hand, PGPR directly strengthen the ability of plants to absorb water and mineral nutrients by secreting metabolites such as organic acids and siderophores and regulating root architecture, thereby alleviating osmotic imbalance and nutrient deficiency caused by stress [113]. On the other hand, PGPR, as key biological “elicitor”, can systematically activate the endogenous defense system of plants, including increasing the activities of antioxidant enzymes such as superoxide dismutase (SOD) and peroxidase (POD), to remove excessive reactive oxygen species (ROS), reduce membrane lipid peroxidation damage, and induce the accumulation of osmotic regulators such as proline to maintain cell homeostasis [114]. More importantly, PGPR can reshape the rhizosphere microenvironment, and its metabolites can be used as signal molecules or nutrient substrates to attract and enrich other beneficial microorganisms, thus forming a more resistant rhizosphere microbial community [115].

Although the great potential of PGPR has been verified in many crops, there are still significant gaps in its research and application in vegetable crops. At present, the efficient PGPR resources isolated and identified from the rhizosphere of vegetable crops are still relatively limited, mainly concentrated in several common genera such as *Pseudomonas*, *Bacillus*, and *Enterobacter*, and there is a unique core microbiome that has not been fully excavated. In recent years, technologies such as high-throughput sequencing and culturomics have overcome the restrictions of traditional isolation and culture techniques, revealing that under drought, high-salt, and other stresses, the rhizosphere metabolic spectrum of vegetable crops undergoes specific changes and drives the directional enrichment of stress-tolerant functional microorganisms [116]. Therefore, the directional screening of PGPR from adversity has become an effective strategy for obtaining highly active strains. However, the exploration of microbial resources in extreme environments still faces the challenges of difficult sample acquisition and low culturability of strains; therefore, the development new in situ culture and fidelity identification techniques is urgent.

At present, the biggest obstacle to the application of PGPR from laboratory to field is effect instability. The field growth-promoting and stress-resistant effects of strains with excellent performance under controllable conditions are often greatly reduced due to weak colonization ability, soil indigenous microbial competition, and fluctuations in environmental factors. In theory, the compound microbial agent can provide more stable and comprehensive benefits than a single strain through functional synergy and niche complementarity between the flora [117]. For example, the combination of strains with ACC deaminase activity (alleviating stress ethylene) and phosphate-solubilizing nitrogen-fixing bacteria (promoting nutrition) has shown potential in theory. However, in practice, the synergistic effect of synthetic microbial communities (SynComs) is not inevitable, and blind combination may even lead to antagonism. The fundamental reason for this is that there is a lack of systematic understanding of the interaction mechanism of the complex system of the “plant–inoculant–indigenous microbiome–soil environment”. Previous studies have focused on the phenotypic response of plants after inoculation, but little is known about the true colonization dynamics of PGPR in the rhizosphere, the interaction network with indigenous flora, and the cascade effect on soil biogeochemical cycles.

In order to improve upon the abovementioned limitations, future research is required to take a double-track parallel approach in terms of mechanism depth and application breadth. At the mechanism level, the research paradigm of system biology must be adopted. The integration of metagenomics, metabolomics, transcriptomics, and microbial imaging technology can not only analyze the structure and function of the core microbiome in the rhizosphere of cucurbits under stress but also reveal the molecular dialog mechanism in situ between PGPR and plants (such as quorum sensing and metabolite exchange), as well as the interaction network between flora. This helps us shift from the description of “who is there” to the functional understanding of “what they are doing” and “how they interact”, laying a theoretical foundation for the rational design of synthetic flora.

At the level of application transformation, future directions should focus on personalization and precision. First, it is necessary to promote microbiome-oriented breeding, screen and cultivate vegetable crop genotypes that are better at recruiting and maintaining beneficial rhizosphere flora, and achieve two-way selection between plants and microorganisms. Secondly, the rational design and assembly of functional modules (such as stress sensing and specific metabolite synthesis) of PGPR with excellent colonization or chassis-promoting ability should be achieved by means of synthetic biology to create a new generation of intelligent engineering bacteria. Finally, the field management strategy must be upgraded from simple microbial inoculation to microbial ecosystem management. This includes the development of microbial agent delivery systems based on new materials such as biochar and hydrogel to protect cell viability and the pre-adjustment of soil microenvironment through agronomic measures (such as reasonable water and fertilizer management) to create favorable conditions for the successful colonization and function of inoculated bacteria.

## Figures and Tables

**Table 1:** Growth-promoting mechanisms and effects of plant growth-promoting rhizobacteria for alleviating abiotic stresses in vegetable crops.

Abiotic Stress	Crop	Plant Growth-Promoting Phizobacteria	Function	Reference
Drought	*Brassica campestris* L.	*Flavobacterium* sp. GJW24	Regulating stomatal closure and up-regulating the expression of drought stress-responsive genes such as *EXLB1*, *DREB2A*, *TIFY3a* and *CSD3*.	[78]
	*Solanum* *lycopersicum* L.	*P. aeruginosa* N5.12 and N21.24	Increasing photosynthetic pigments, proline, betaine and other substances, as well as increasing the expression of the abscisic acid synthesis rate-limiting enzyme gene *NCDE1* and the pyrroline synthase gene *P5CS*.	[82]
	*Capsicum annuum*	*Enterobacter cloacae* AAUSR23, *Pseudomonas fluorescens* AAULE41, and undetermined AAULE51	Producing IAA and extracellular polysaccharides.	[83]
	*Brassica juncea* L.	*Enterobacter hormaechei, Acinetobacter* sp., and *Pantoea dispersa*	Enhancing osmotic regulation, reactive oxygen species (ROS) detoxification and carbon and nitrogen metabolism.	[84]
	*Cicer arietinum* L.	*Bacillus amyloliquefaciens* NBRISN13 and *Pseudomonas putida* NBRIRA	ACC deaminase activity, mineral solubilization, hormones production, biofilm formation, siderophore activity.	[85]
Salinity	*Brassica juncea* L.	*Pseudomonas fluoresnes* UM270	Modulating antioxidant activity; reducing oxidative stress response and IAA production.	[74]
	*Cucumis sativus* L.	*Bacillus subtilis* C8	Siderophore and IAA production.	[88]
	*Brassica juncea* L.	*Pseudomonas fluorescens* NAIMCC-B-00340 and *Azotobacter chroococcum* Beijerinck 1901	ACC deaminase activity; maintained reactive oxygen species homeostasis.	[90]
Heat	*Lactuca sativa* L.	*Klebsiella* sp. GRB10	Regulating stomatal movement, improving photosystem II efficiency, and reducing water consumption.	[93]
	*Solanum lycopersicum* L.	*Bacillus cereus* KTES	ACC deaminase and extracellular polysaccharides.	[94]
Cold	*Solanum lycopersicum* L.	*Bacillus cereus* AR156, *Bacillus subtilis* SM21, and *Serratia* sp. XY21	Promoting the accumulation of soluble sugars, proline, and osmotic proteins in tomato; enhancing the antioxidant defense system; and reducing osmotic stress in plant cells.	[95]
Heavy metal	*Solanum nigrum* L.	*Acinetobacter pittii*	Enriching groups related to plant growth promotion and Cd mobilization in rhizosphere soil.	[75]
	*Solanum lycopersicum* L.	*Pseudomonas fluorescens* A506	Enhancing the antioxidant activities of catalase, peroxidase, and superoxide dismutase; improving physiological traits such as chlorophyll and proline contents.	[76]

## Data Availability

Data are contained within the article.
